# Influence of Copper Dose on *Mycobacterium avium* and *Legionella pneumophila* Growth in Premise Plumbing

**DOI:** 10.1021/acsestwater.5c00959

**Published:** 2025-10-10

**Authors:** Rania E. Smeltz, Fernando A. Roman Jr, Thomas Byrne, Rachel Finkelstein, Yang Song, Amy Pruden, Marc A. Edwards

**Affiliations:** † Department of Civil and Environmental Engineering, 1757 Virginia Tech, Blacksburg, Virginia 24061, United States; ‡ Department of Microbiology, University of Alabama at Birmingham, Birmingham, Alabama 35294, United States; § Department of Genetics, Bioinformatics, and Computational Biology, Virginia Tech, Blacksburg, Virginia 24061, United States; ∥ AECOM, 3101 Wilson Boulevard, Arlington, Virginia 22201, United States; ⊥ Utilities Department, Town of Cary, 316 N. Academy Street, Cary, North Carolina 27513, United States

**Keywords:** Legionella, mycobacteria, opportunistic pathogens, copper, drinking water, cross-linked polyethylene pipes

## Abstract

Effects of copper at 0, 4, 30, 250, or 2000 μg/L on microbial communities were examined over an 11 month dosing period using triplicate 120 mL water heater microcosms with PEX-b pipes containing mature biofilms to simulate premise plumbing. Effluent total cell counts (TCCs) and *Mycobacterium avium* peaked at 250 μg/L, reflecting the dual role of copper as a nutrient and antimicrobial. TCCs and *M. avium* were relatively consistent among replicate microcosms at each dose, but *Legionella pneumophila* (*Lp*) diverged among biological triplicates at 250 μg/L, consistently producing high culturable *Lp* (average 2.5 log MPN/mL) in one microcosm and low/nondetectable levels in the other two. Repeated cross-inoculations and a reinoculation failed to normalize the microbial community composition across 250 μg/L and other triplicate microcosms. 16S rRNA gene amplicon sequencing revealed that the 250 μg/L replicate with a high *Lp* was characterized by a distinct microbial community composition relative to the two replicates. At 2000 μg/L copper, microbial diversity and TCCs initially decreased, but then TCCs subsequently increased and ultimately were not statistically different from the 250 μg/L microcosms. This study provides insight into mechanisms underlying nonlinear effects of copper dosing when applied as a disinfectant to premise plumbing for opportunistic pathogen control.

## Graphical abstract

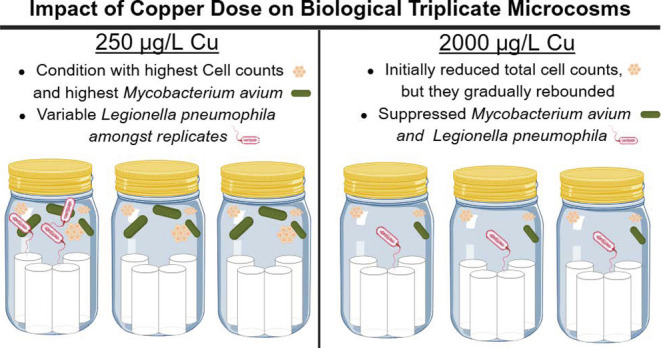

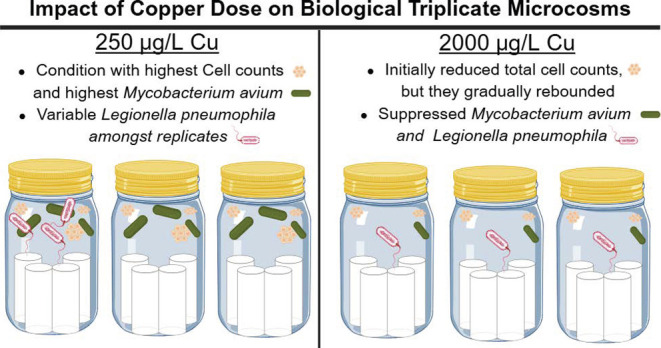

## Introduction

Copper is of interest as a disinfectant for the control of opportunistic pathogen growth in premise (i.e., building) plumbing. Copper can be released into drinking water from corrosion of copper alloy pipe or intentionally dosed for this purpose.^1^ Broadly, the efficacy of copper as an antimicrobial can be dependent on water chemistry, the physiology of the target microbes, biofilm characteristics, water flow patterns, levels of other disinfectants (e.g., chlorine), and other factors.^2,3^ Further, copper has been found to sometimes act as a nutrient to drinking water microbes at low concentrations and an antimicrobial at high concentrations,^2^ suggesting that suboptimal doses could stimulate growth, rather than death, of pathogens. Thus, there is an array of factors to consider in applying copper as a premise plumbing disinfectant.

*Mycobacterium avium* and *Legionella pneumophila* (*Lp*) are two key opportunistic pathogens of concern that are prone to growth in premise plumbing.^2^ Prior field surveys of *Legionella* and *Lp* occurrence in drinking water suggest antimicrobial thresholds for copper of >50 μg/L,^4^ > 400 μg/L,^5^ or >1055 μg/L.^6^ However, other studies have reported contradictory results, suggesting positive effects on *Legionella* and *Lp* growth at concentrations >500 μg/L,^7^ or *Lp* persistence at concentrations >2000 μg/L.^8^ *M. avium* has been shown to be inactivated by high levels of copper in warm premise plumbing environments,^9,10^ but *M. avium* and other nontuberculous mycobacteria (NTM) are generally considered more resilient to copper than *Legionella*.^10,11^ These prior studies suggest that copper, if present at sufficiently high concentrations, could sometimes effectively control both NTM and *Lp*, which is the ideal case. Nonetheless, many existing strategies for controlling opportunistic pathogens in drinking water have limitations and, in some instances, may inadvertently promote pathogen proliferation.^11,12^ Controlled studies are needed to resolve discrepancies in the efficacy of copper as a disinfectant and to gain a deeper mechanistic understanding needed to improve its application for simultaneous reduction of multiple opportunistic pathogens.

To gain insight into the effects of copper on *Lp* and NTM control in hot water plumbing systems, specifically at temperatures that support opportunistic pathogen growth in premise plumbing, we previously conducted a series of studies at both pilot- (spanning 3 years) and microcosm- (spanning 198 days) scales using the same local Blacksburg water supply.^9,13,14^ These studies demonstrated how a complex array of phenomena, including anode rod corrosion, associated hydrogen evolution, and pH shifts, can confound the antimicrobial action of copper. Similar confounding effects were likely at play in prior field-scale research but were generally not considered in the interpretation of *Lp* occurrence trends.^2^ Comparatively, another relatively long-term study (12 months) investigating opportunistic pathogens in water heaters was only able to detect NTM, and not *Legionella* spp., *Lp*, or *Pseudomonas aeruginosa* at any point in the experiment,^15^ highlighting challenges in the simultaneous study of *Lp* and NTM at pilot scale, especially over extended time periods. In another prior microcosm-scale study, the effect of a 0.8–5 mg/L total Cu dose on *Lp* culturability was examined over a 4 week period using a synthetic water at 36 °C.^16^ That work found that different *Lp* strains reacted differently to copper. Furthermore, environmental isolates derived from biofilm in a hot water system exhibited greater resistance to copper compared to the clinical strains tested, suggesting that the environmental isolates (which had a significantly higher expression of the copper resistance gene copA) were better adapted to copper.^16^ Finally, a 76 week microcosm-scale study examined the impact of different materials (including pipe coupons) on *Lp* and NTM, finding that high concentrations of copper (≈10 mg/L) suppressed established NTM and *Lp* and lowered their concentrations in the microcosms.^17^

The objective of this study was to assess the effects of a wide range of copper concentrations on *M. avium* and *Lp* in premise plumbing systems colonized by both organisms. Fifteen 120 mL water heater microcosms equipped with PEX-b pipes containing mature biofilms (>3.5 years old) and colonized with both *Lp* and *M. avium* ^9^ were acclimated to Blacksburg tap water over a 6 month period and subsequently divided into biological triplicate microcosms subject to copper dosing at 0, 4, 30, 250, or 2000 μg/L (as total copper) for 11 months. Microbial total cell counts (TCCs) were monitored via flow cytometry; *M. avium* was measured via droplet digital polymerase chain reaction (ddPCR), and *Lp* was measured both via ddPCR and Legiolert. 16S rRNA gene amplicon sequencing was applied to profile microbial communities and provide insight into microbial ecological factors mediating the effects of copper. The goal was to assess doses at which copper acted as a nutrient versus as an antimicrobial for opportunistic pathogens under a warm water temperature regime typical of premise plumbing.

## Materials and Methods

### Premise Plumbing Microcosm Design and Establishment

Fifteen new 120 mL glass microcosms (referred to as “simulated glass water heaters” in previous works)^18^ provided replicable simulation of premise plumbing with mature drinking water biofilm in triplicate. Each replicate microcosm contained two new and two conditioned PEX-b pipe coupons, with conditioned coupons cut from the middle of ∼ 4.6 m recirculating lines of pilot-scale hot water plumbing systems, one that was previously dosed with phosphate and one that received no added phosphate over a >3.5 year experiment.^9,13^ All coupons were 2.5 cm long with a ∼1.7 cm inner diameter. The pilot-scale hot water plumbing systems had been inoculated with two strains of *Lp* serogroup-1 that were isolated from the Quincy, Illinois Veterans Home during a Legionnaires’ Disease outbreak.^19^ Prior to this experiment, the recirculation line biofilms were confirmed to be colonized by both *M. avium* and *Lp*,^9^ serving as an inoculum to the microcosms.

### Microcosm Influent and Water Changes: Acclimation Phase

The water heater microcosms were filled with 110 mL of local tap water (Blacksburg, VA) treated by a granular activated carbon filter feeding the recirculating pipe rig from which the pipe coupons were extracted. The influent water was collected in batches at 5 week intervals and stored at 4 °C. On the day before a water change, 2 L of the water was collected and heated to 37 °C overnight, then adjusted to pH 6.70 ± 0.05 using H_2_SO_4_, before addition to the microcosms. The microcosm bulk water was changed 2× weekly by replacing 75% of the bulk water volume (∼82 mL) in each microcosm with new influent to match the 3.2 day hydraulic retention time of the pilot-scale plumbing system. The 3.2 day hydraulic retention time was selected to simulate common residential hot water usage in the United States.^13^ The microcosms were maintained at 37 °C, representing the low end of a water heater set point, and gently mixed on a shaker table at 100 rpm. The microcosms were acclimated for 6 months under these conditions prior to the commencement of copper dosing.

### Cross-Inoculation of the Microcosms

A month into the acclimation phase, the effluent from each microcosm was cross-inoculated with the aim of establishing baseline *Lp* and microbial populations across the 15 replicates. This was achieved by collecting the effluent (75% bulk water volume) from each microcosm combining it into a common reservoir (an autoclaved 2 L polypropylene bottle), vigorously shaking this mixture, returning 25% of the removed bulk water volume to the microcosms with the combined effluent, and replacing the remaining 50% that had been removed with the GAC-treated water. This procedure was repeated over the course of ten sequential water changes, starting a month into the acclimation phase, for 5 weeks thereafter.

### Copper Dosing and Reinoculation

Nearing the end of the 6 month acclimation phase, a week before the copper dosing commenced, the microcosms were sampled for culturable *Lp* levels measured by Legiolert tests (IDEXX Laboratories, Westbrook, ME) and TCCs. Based on these measurements, the microcosms were grouped into five sets of triplicate microcosms, so that there was no statistical difference in mean values of TCC or *Lp* among experimental conditions (ANOVA, *p* > 0.05) (Supporting Information, Figure S1). Triplicate microcosms subsequently received one of five dosages of 0, 4, 30, 250, or 2000 μg/L copper over an 11 month period. Copper was dosed directly to the influent using stock solutions prepared with CuSO_4_·5H_2_O. After ∼3.5 months of copper dosing, random testing among the microcosms indicated that *Lp* still had not established in some replicates, and therefore, all microcosms were reinoculated once more. This was achieved using water from the same pilot-scale water heater from which the microcosm pipe coupons were originally derived. The inoculation water was mixed with the normal influent water to target ∼50 MPN/mL culturable *Lp* in each microcosm.

The influent water preparation protocol was changed throughout the 6 month acclimation (experimental months 0–6) and 11 month copper dosing (experimental months 6–17) phases of the experiment in an attempt to establish a replicable response of *Lp* to copper. Modifications included: (1) passing GAC-filtered water through a ferric oxide filter to decrease phosphate levels to <0.05 mg/L, followed by filter-sterilizing the water using a 0.22 μm pore size mixed nitrocellulose ester membrane (Whatman, Maidstone, United Kingdom) during the experimental months 0–8; (2) dosing GAC-treated influent with phosphate throughout the copper dosing phase to achieve 5 μg/L as P during experimental months 6–17; (3) no longer sterilizing the influent or treating it with a ferric oxide filter during experimental months 8–17; and (4) dosing ferric pyrophosphate (100 μg as Fe) after week 35 of the copper dosing phase during experimental months 14–17. Modifications represented failed attempts at achieving consistent *Lp* growth across the microcosms: (1) phosphate and background microbes were initially minimized (months 0–8) to match the design of the source rig and avoid confounding influences; (2) phosphate supplement (months 6–17) to eliminate a possible nutrient limitation; (3) sterilization and ferric oxide treatment were discontinued (months 8–17) to allow natural phosphate and background microbes; and 4) ferric pyrophosphate was introduced (months 14–17) to create a bioavailable iron source.

### Microbial Analysis

Effluent collected from the microcosms during biweekly water changes was subject to microbial analysis. TCC was measured on a BD Accuri C6 (BD Bioscience, Franklin Lakes, NJ) using SYBR Green I dye to stain total (intact + damaged) cells.^20–22^ Gating used the Eawag FL1-A (emission filter 533/30) vs FL3-A (emission filter 670 LP) template for drinking water, with 50 μL of each sample analyzed at medium speed (35 μL/min) and with an acquisition threshold set to 800.^22^ Legiolert was used to enumerate culturable *Lp* throughout the study, subjecting 1.0 mL of sample bulk water to the nonpotable procedure per the manufacturer’s instructions.

During regular water changes, ∼82 mL of influent or effluent water from each microcosm was filtered through a 0.22 μm mixed-cellulose ester membrane filter (MilliporeSigma, Burlington, MA), and the filter was subjected to DNA extraction using a FastDNA SPIN kit (MP Biomedicals Inc., Solon, OH). These filtering events and the corresponding DNA for microbial analyses were obtained during three consecutive routine water changes (3–4 days apart) during the final (11th) month of copper dosing (17th month of the experiment).

### Droplet Digital PCR Analysis

DNA extracts were analyzed for opportunistic pathogens using a QX200 ddPCR instrument (Bio-Rad, Hercules, CA) (Supporting Information, Table S1). These assays, targeting the *Lp* specific *mip* gene and *M. avium* 16S rRNA gene, had previously been optimized for qPCR^23^ and were adapted for ddPCR in this study. Thermal gradients, ranging from 50 to 60 °C, were conducted on each assay to determine the optimal annealing temperature (Supporting Information, Table S1) to separate negative and positive droplets. Each sample was analyzed in a technical triplicate for *Lp* and *M. avium*. Reactions were prepared using a total volume of 22 μL per well, with 20 μL in each well used for analysis. PCR amplification was carried out on a C100 Touch Thermal Cycler (Bio-Rad) (thermocycling conditions listed in Supporting Information, Table S1). Each 96-well plate included three no-template controls using molecular-grade water as well as three positive controls containing synthetic gene fragments (gBlocks, Integrated DNA Technologies, Coralville, IA) corresponding to the targeted gene regions. A sample was classified as positive if a minimum of three positive droplets were detected in at least two of the three of the technical replicates.^24^ Only data from wells generating >10,000 accepted droplets were included in the analysis.

### 16S rRNA Gene Amplicon Sequencing and Data Analysis

To analyze the microbial composition of each microcosm, 16S rRNA gene amplicon sequencing was carried out on the DNA extracts. Amplicon sequence reads targeted the V4–V5 hypervariable region of the 16S rRNA gene by using the 515f/926r primer set. Samples were sequenced on a MiSeq V3 600 cycle run, yielding an average of 119,910 reads per sample (*n* = 83, SD = 69,156). Reads were imported, and subsequent analysis was carried out using DADA2 (v1.18.0)^25^ in R (v 4.0.3).^26^ Reads were filtered, trimmed, and paired before exact amplicon sequence variants (ASVs) were generated and the chimeras removed. Taxonomy of ASVs was assigned using RDP Train Set 18.^27^ ASVs were rarefied to the minimum sequencing depth of 10,544.^28^ Multivariate homogeneity was checked with the betadisper R function (*p* > 0.05) before conducting permutational multivariate analysis of variance (PERMANOVA) with Bray–Curtis distance matrices using the vegan R package (v 2.6.4).^29^ Linear mixed-effect models were created on *M. avium* and *Lp* ddPCR measurements, controlling for the three consecutive samplings of each microcosm in the final month of the experiment using the lme4 R package (v 1.1-36).^30^ ANOVA was run on the linear mixed-effect models to assess the impact of copper dosage on the Shannon and Simpson diversity (calculated with vegan) using the R stats package (v 4.4.2).^26^ Tukey’s HSD was used as a posthoc test to ANOVA to assess the impact of each copper dosage using the multcomp R package (v 1.4-28).^31^ Wald’s test was used for the log2 fold change between the 250 μg/L Cu condition microcosms using the DESeq2 R package (v 1.46.0).^32^ A paired *t*-test controlling for repeated measures was used to assess differences in TCC before and after copper dosing using the R stats package (v 4.4.2).^26^

### Data Availability

The 16S rRNA gene amplicon sequencing gathered from the microcosms in this experiment has been uploaded to the National Center for Biotechnology Information Sequence Read Archive database under Accession no. PRJNA1229645.

## Results

### Total Cell Counts

The effluent mean TCC for all of the microcosms during the end of the acclimation phase (month 6), before copper addition, was 3.18 × 10^5^ cells/mL (SD = 4.16 × 10^3^) (Figure 1, Supporting Information, Figure S1b). In the short term after copper dosing began (1–3.5 months post copper dosing), the TCC indicated growth at 250 μg/L. As the experiment progressed, the three replicates dosed with 250 μg/L copper consistently produced the highest cell counts. Replicates dosed with 2000 μg/L copper initially experienced a 0.66 log reduction (a 78% decrease from their final predosing concentration), resulting in the lowest TCCs observed in the study. But by the end of the 11 month copper-dosing period, the mean TCC at 2000 μg/L copper gradually recovered and eventually surpassed those at doses ≤30 μg/L and was not significantly different than at 250 μg/L (Tukey’s HSD *p* < 0.05) (Figure 1, Supporting Information, Table S2). In fact, the TCC steadily increased with time in the 2000 μg/L microcosms, over a time period spanning both before and after the reinoculation attempt that occurred 3.5 months into the copper dosing phase (Figure 1, Supporting Information, Figure S2).

**Figure 1:**
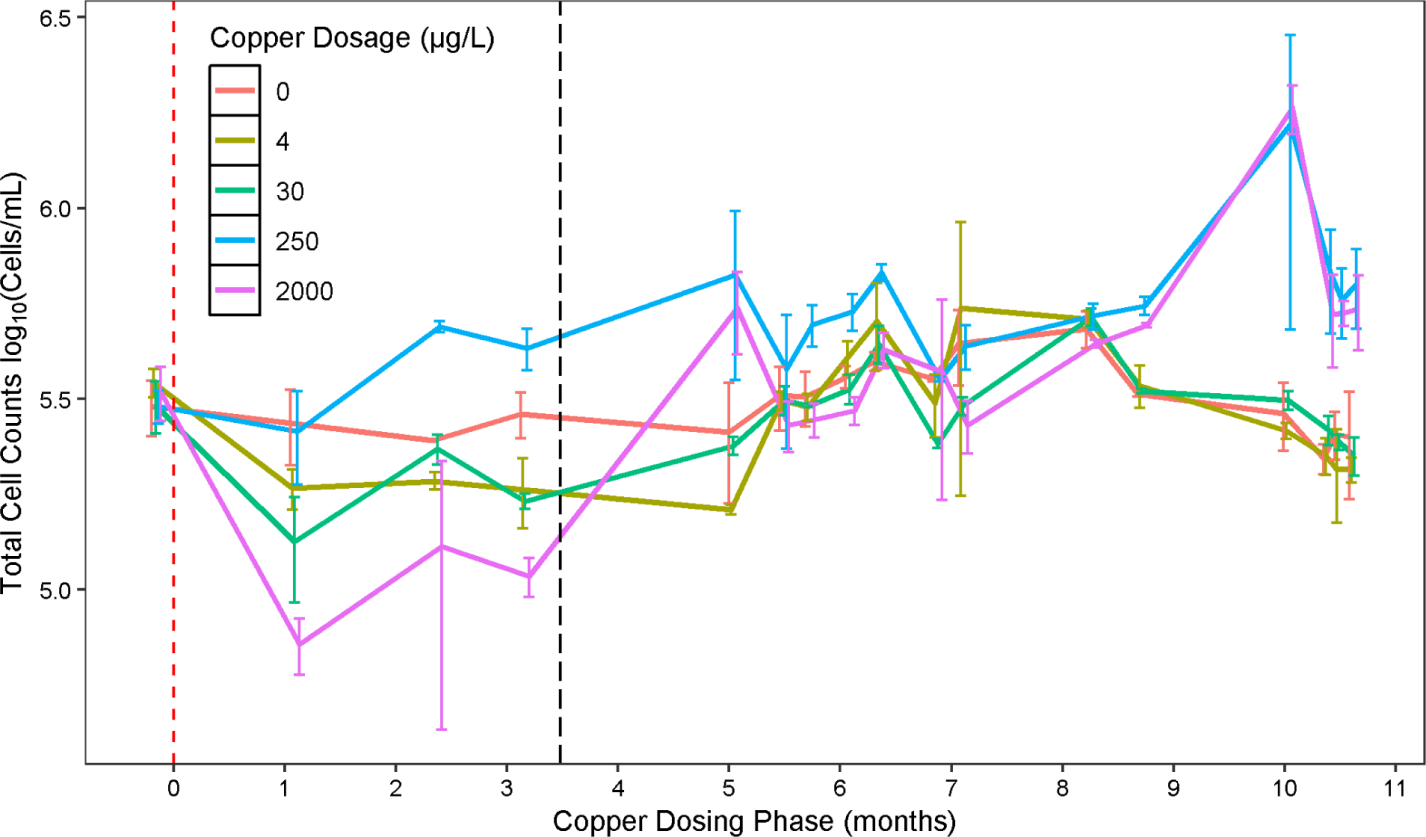
Mean TCCs (cells/mL) in microcosm bulk water effluent over the course of the copper-dosing period. Error bars are the standard deviations of biological triplicate microcosms. Lines connect the data points to guide comparison and are colored by the copper concentration. The first measurement (time = −0.25) occurred at the end of the acclimation phase, a week before copper dosing began (time = 0 represented by the red dashed vertical line). The black dashed vertical line at ∼3.5 months represents the point at which all microcosms were reinoculated with *Lp*. Time points have a set jitter to avoid overlapping data points.

### *M. avium* Response to Copper in the Microcosms

When tested over three sequential samplings in the final (11th) month of the copper-dosing period (month 17 of the overall experiment), significant differences in *M. avium* gene copies/mL were found in the microcosm bulk water as a function of copper concentration (Linear Mixed-Effect Model ANOVA, *p* < 0.05) (Figure 2). The 250 μg/L condition contained higher concentrations of *M. avium* compared to other copper dosages (0.55–0.85 logs higher) (Tukey’s HSD, *p* < 0.05, Supporting Information, Table S3). *M. avium* was not detectable in the influent water by using ddPCR, indicating that the increase in the effluent was the result of growth in the microcosms.

**Figure 2:**
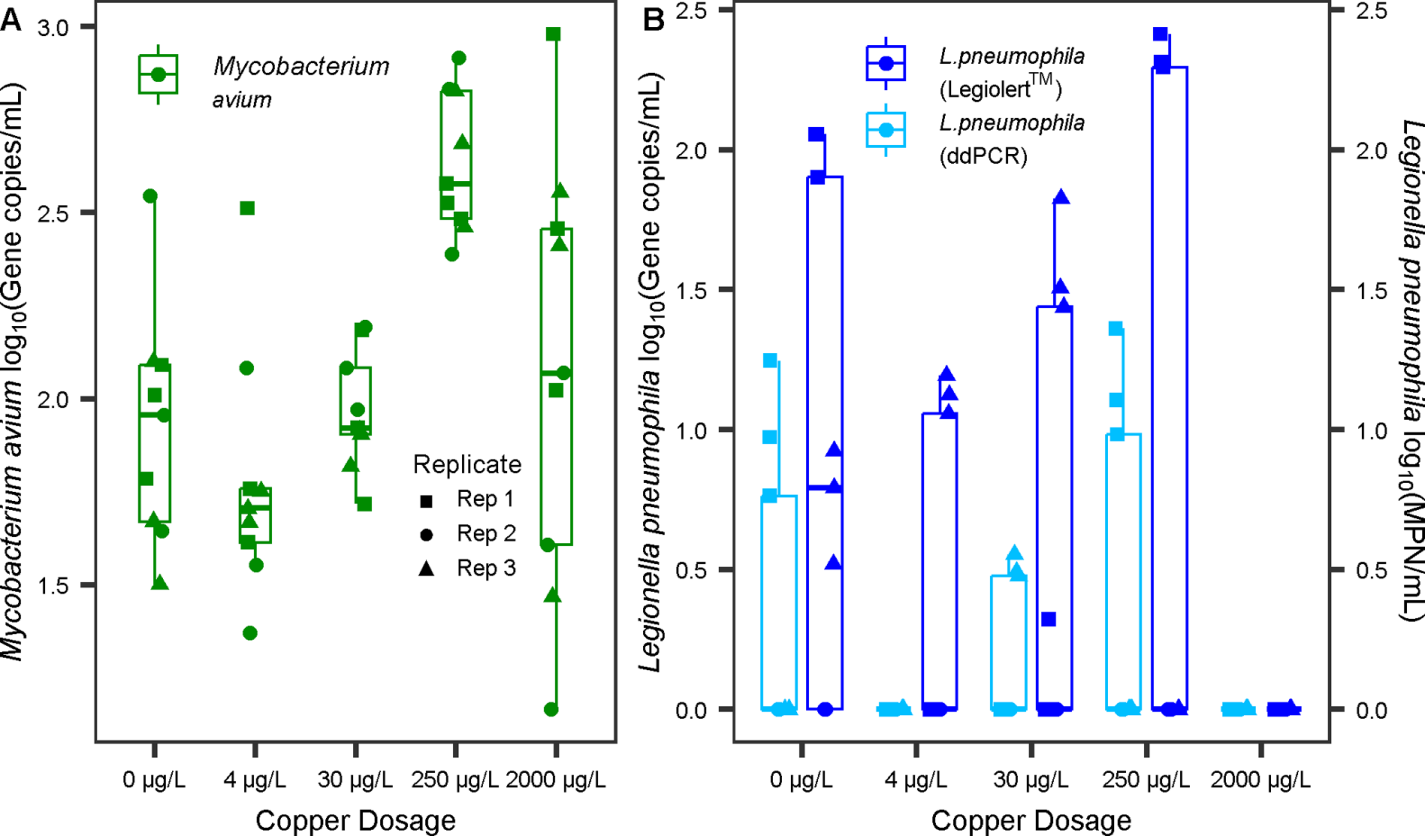
Mean ddPCR technical triplicate gene copies/mL counts of (A) *M. avium* detected through ddPCR and (B) *Lp* detected via ddPCR (mean technical triplicate) and Legiolert across the 15 microcosms over three consecutive samplings in the final (11th) month of the copper-dosing period. For (A), the data consists of 5 copper levels × 3 microcosms × 3 sampling events = 45 *M. avium* data points). For (B), the data consists of 5 copper levels × 3 microcosms × 3 sampling events = 45 culturable (MPN/mL) or 45 ddPCR (gc/mL) *Lp* data points. *M. avium* was detectable in all of the samples. For *Lp*, points plotted at zero (gc/mL or MPN/mL) indicate nondetects for ddPCR (estimated ∼ detection limit of 1.5 gc/mL) and Legiolert (detection limit of 1 MPN/mL for the protocol used).

### *L. pneumophila* Response to Copper in the Microcosms

*Lp* response to copper dosing was confirmed by both ddPCR and Legiolert. On dates when both Legiolert and ddPCR tests were conducted on aliquots of the same sample (*n* = 45), measurements were strongly correlated (*R* ^2^ = 0.85) (*t*-test, *p* < 0.05), but the log ddPCR gc/mL values were typically 0.51 times the log of Legiolert MPN/mL (Supporting Information, Figure S3). *Lp* concentrations did not significantly differ across the microcosms as a function of copper condition (Linear Mixed-Effect Model ANOVA, *p* > 0.05), but both Legiolert and ddPCR measurements indicated a consistent ranking of average concentration across microcosms as follows: 2000 μg/L ≤ 4 μg/L < 30 μg/L < 0 μg/L < 250 μg/L. *Lp* was never detectable in the influent used for the microcosm water changes using ddPCR or Legiolert, indicating that the increase in the effluent was the result of growth in the microcosms. It was noted that there was a much wider variance in *Lp* numbers among replicates than there was for *M. avium* (Figure 2). This was most evident for microcosms dosed with 250 μg/L copper, for which one microcosm consistently displayed the highest concentrations of culturable *Lp*, while the second and third replicates produced nondetectable levels of culturable *Lp* over the majority of the sampling events (87% and 73% of events) over the course of the copper-dosing phase of the experiment (Figure 3). *Lp* levels quantified via Legiolert ranged from 8 MPN/mL to 1460 MPN/mL for the first replicate microcosm in the 250 μg/L Cu condition (Figure 3). The stark differences between these microcosms were maintained even though they had been cross-inoculated ten times during acclimation to establish the same baseline microbial community prior to copper dosing, reinoculated ∼ 3.5 months into the copper dosing phase, and subjected to four adjustments to the water chemistry.

**Figure 3:**
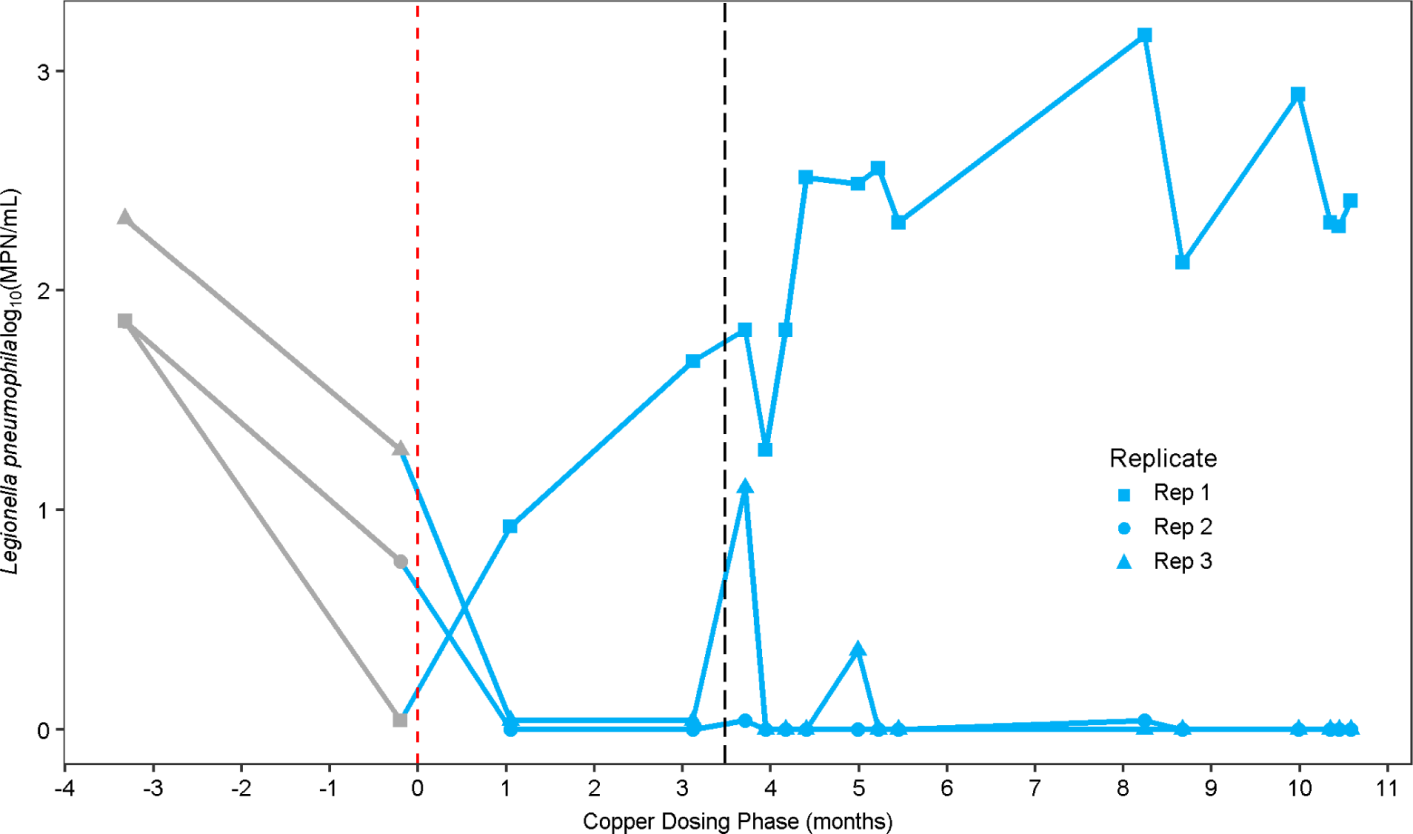
*Lp* MPN/mL, determined via Legiolert, was among the three replicate microcosms dosed with 250 μg/L copper that displayed divergent behavior over the 11 month copper-dosing period. The vertical dashed line at ∼3.5 months represents the point at which all microcosms were reinoculated with *Lp*. Time 0, shown with the red dashed vertical line represents when the copper dosing began. Points before 0 months represent culturable *Lp* in the microcosms prior to copper dosing during the acclimation phase. Culturable *Lp* concentration measured with time for the microcosms receiving other copper concentrations can be found in Supporting Information, Figure S4. Legiolert detection limit was 1 MPN/mL. Points plotted at an MPN/mL of zero indicate nondetects for culturable *Lp*.

Consistent with the Legiolert results, replicates 2 and 3 of the 250 μg/L copper condition consistently yielded undetectable gene copies/mL of *Lp*, while up to 22 gene copies/mL were measured in replicate 1 (Figure 2).

### 16S rRNA Gene Amplicon Sequencing

16S rRNA gene amplicon sequencing allowed a comparison of the microbial community profiles across the microcosms and influent water (Figure 4). The microbial communities were significantly different from each other as a function of copper concentration (PERMANOVA, *p* < 0.05) (Figure 5). Copper doses of 0, 4, and 30 μg/L produced a similar microbial profile in terms of the ten most abundant phyla from each replicate microcosm, with a marked shift in composition in the 250 and 2000 μg/L conditions (Figure 4). Of note, the microcosms dosed with 0, 4, and 30 μg/L copper were mostly comprised of *Proteobacteria*, while the reactors dosed with 250 μg/L copper contained relatively equal proportions of *Proteobacteria* and *Gemmatimonadetes*. Microcosms dosed with 2000 μg/L were primarily dominated by *Bacteriodetes* and *Proteobacteria* (Figure 4). Within each condition from 0 to 250 μg/L, the microbial communities appeared relatively consistent among the three replicates. However, there was marked variation in the microbial community among the 2000 μg/L condition replicates (Figure 5). Copper concentration had a significant effect on Shannon diversity and Simpson diversity (ANOVA, *p* < 0.05) (Figure 6).

**Figure 4:**
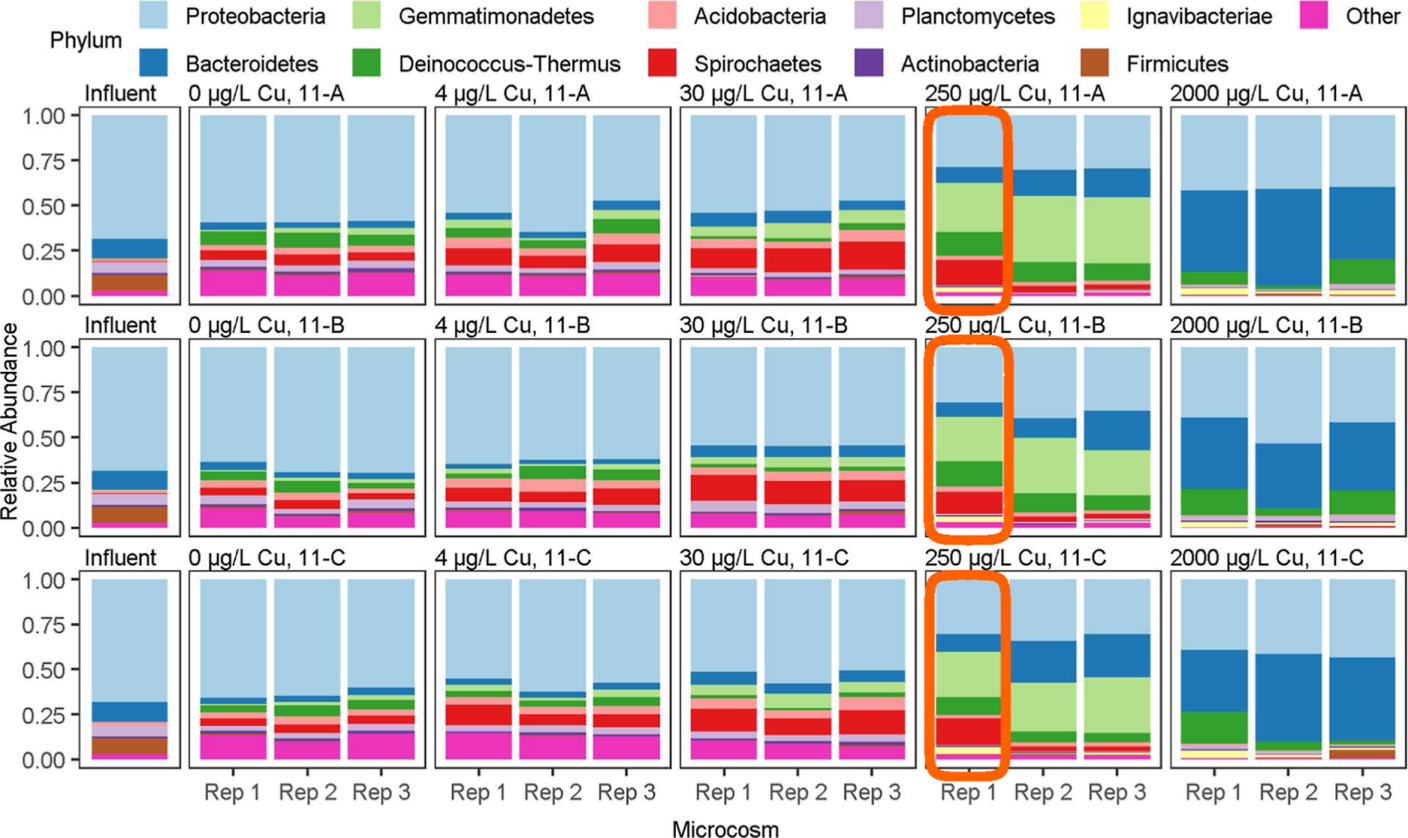
Relative abundances of each of the top ten most abundant phyla over all ASVs in each replicate microcosm grouped by copper concentration and sampling date. The orange boxes indicate the 250 μg/L copper replicate that consistently displayed the highest concentrations of *Lp*. The influent [collected at the start of month 10 for water changes and filtered during month 11 (*n* = 1)] represents the water used throughout three sequential sampling events during water changes carried out in the final (11th) month of the copper-dosing period. 11-A/B/C represent the first (A), second (B), and third (C) sequential water changes.

**Figure 5:**
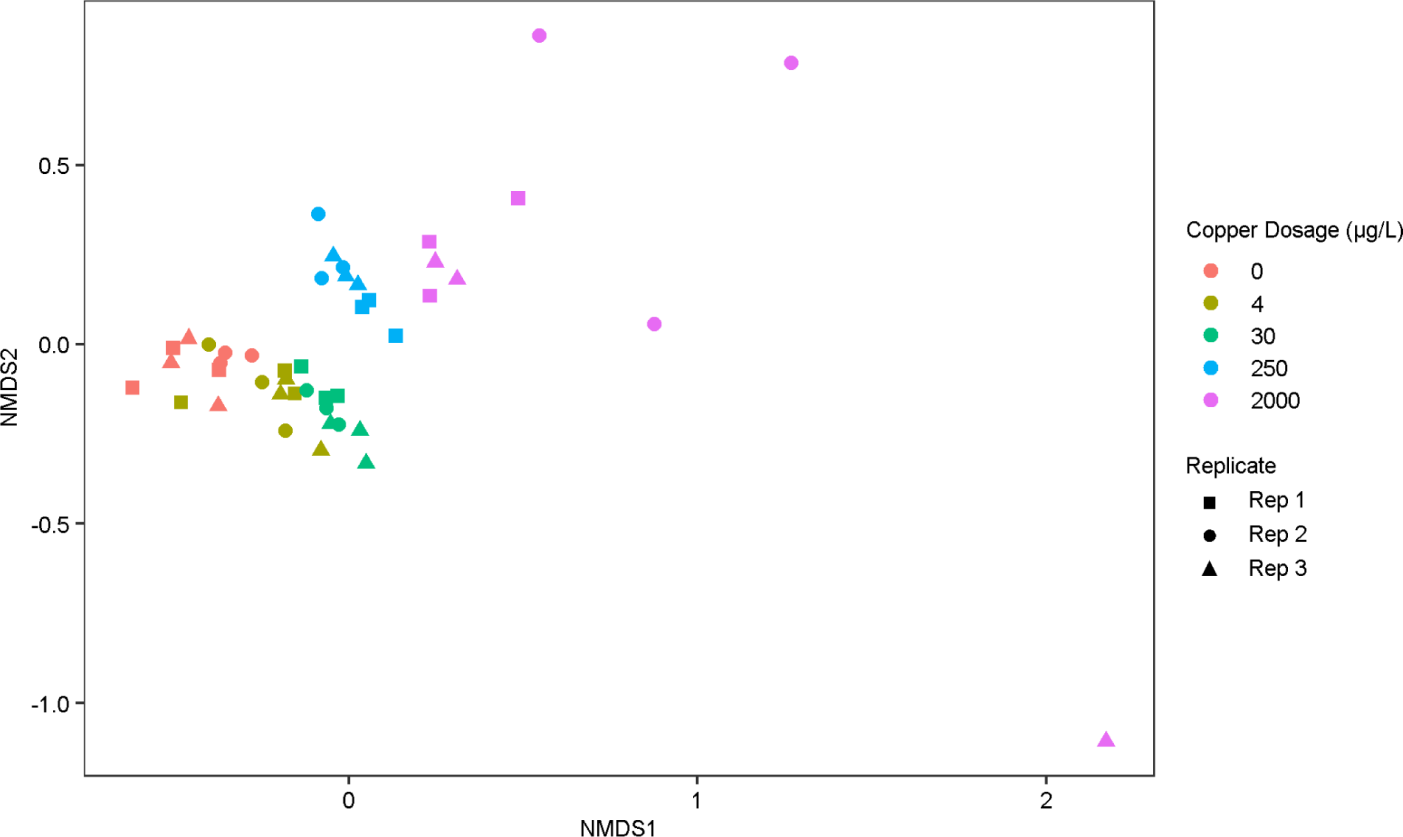
Bray–Curtis-based nonmetric multidimensional scaling of taxa from effluent water of microcosms dosed with 0, 4, 30, 250, and 2000 μg/L of copper over an 11 month copper-dosing period (stress = 0.08836). The data consist of 5 copper levels × 3 microcosms (per copper level) × 3 sampling events = 45 data points.

**Figure 6:**
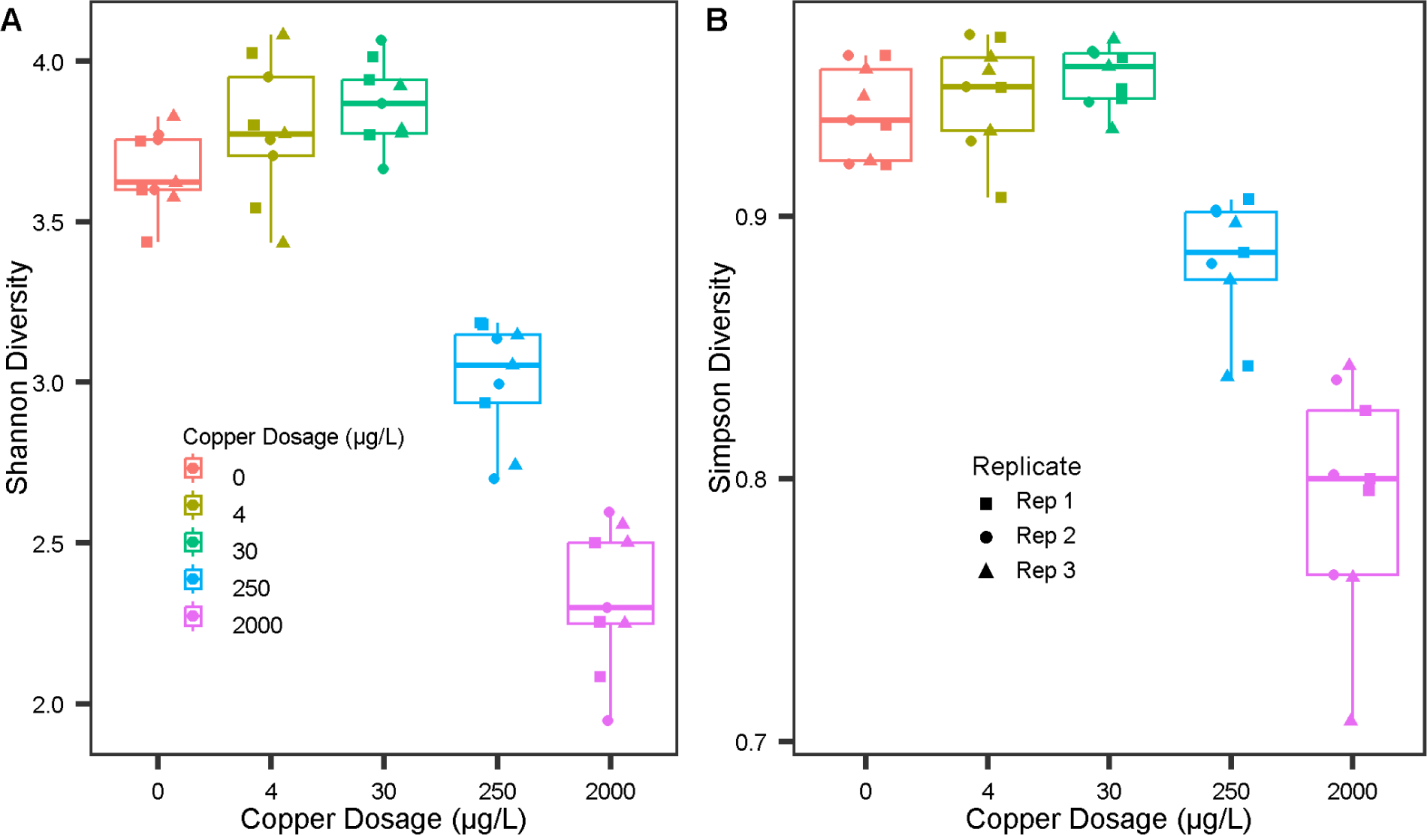
Shannon and Simpson diversity index of replicate microcosms on the final three sampling dates, conducted during the 11th month of the copper-dosing period, grouped by copper condition. The data consists of 5 copper levels × 3 microcosms (per copper level) × 3 sampling events = 45 data points.

The microbial community profile of the 250 μg/L microcosm that consistently had the highest level of *Lp* was found to be dominated by the same top ten phyla, but differences in relative abundance stood out (Figure 4, highlighted in orange boxes). Specifically, this microcosm was significantly enriched in *Ignavibacteriae* and depleted in *Chlamydiae* and *Bacteroidetes* (Wald’s test, *p* < 0.05) (Figure 4, highlighted in orange boxes). Beta diversity analysis, however, did not reveal an obvious difference in the microbial community composition of this replicate beyond the variation seen for replicates of other copper concentrations (Figure 5).

Relative abundances of individual classes of bacteria were further compared across microcosm conditions, revealing some notable differences as a function of the copper concentration (Figure 7). For example, *Proteobacteria* were higher in the 2000 μg/L condition than in the 250 μg/L condition, while *Firmicutes* indicated little change across copper dosages (Figure 7). *Gemmatimonadetes* were enriched from 0 to 250 μg/L but dramatically decreased at 2000 μg/L (Figure 7). *Deinococcus-Thermus* exhibited a slight decrease in relative abundance from 250 to 2000 μg/L (Figure 7). *Chloroflexi*, *Acidobacteria*, and *Actinobacteria* markedly decreased with increasing copper content (Figure 7).

**Figure 7:**
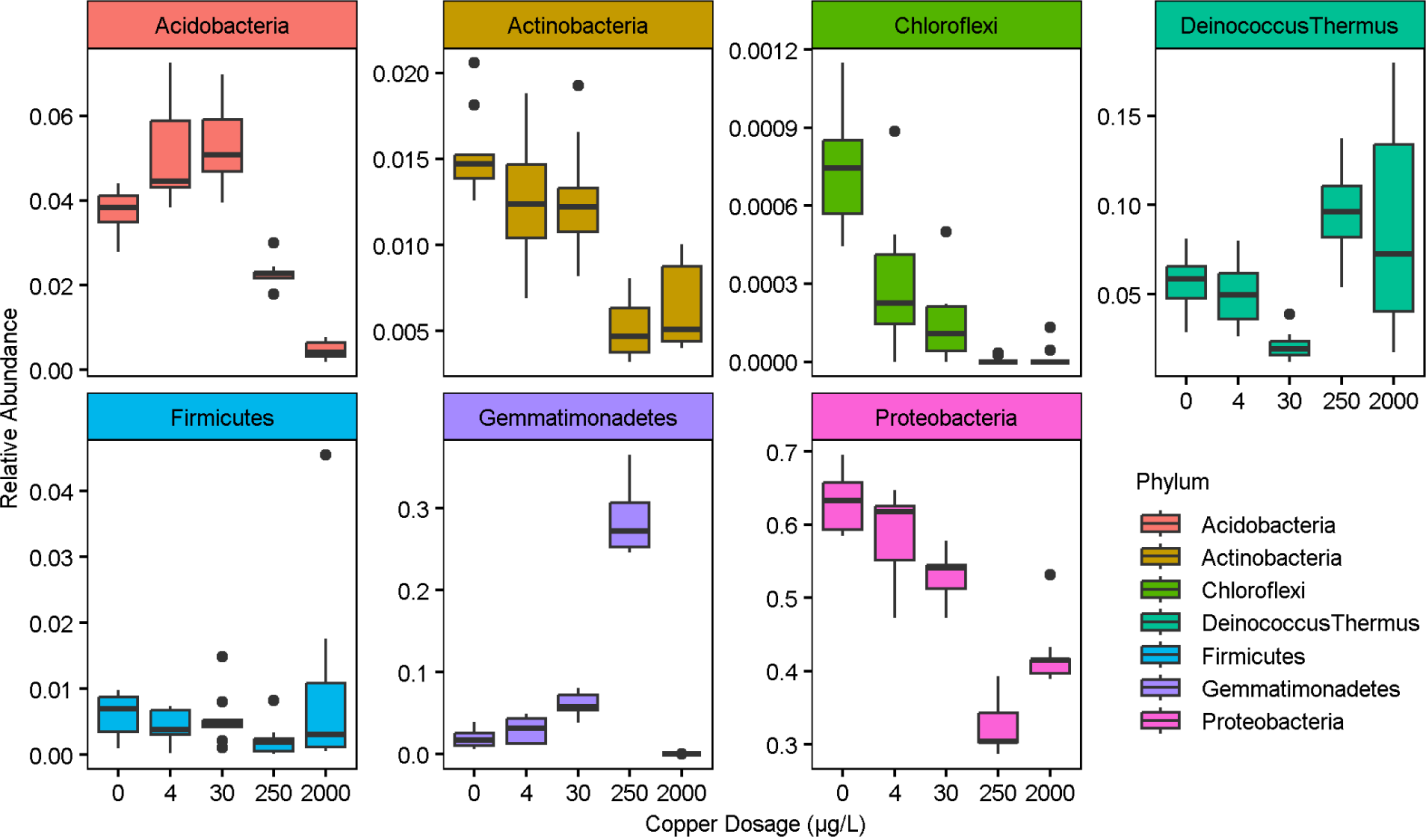
Relative abundance of select phyla across copper dosages in each replicate over the three sequential sampling events carried out in the final (11th) month of copper dosing.

## Discussion

This study reveals apparent hormetic effects of copper on premise plumbing microbiomes, i.e., acting as a nutrient at low concentrations and as an antimicrobial at higher concentrations. TCCs provided an indicator of the effects on bacterial communities at large, revealing some surprising patterns over the course of this near year-long experiment. While TCCs initially decreased markedly when the highest dose of 2000 μg/L was commenced, as one would expect, over the course of subsequent months, the microbial community adapted such that TCCs were as high as 250 μg/L by the end of the experiment. Further, TCC levels in the 2000 μg/L condition had not yet plateaued by the end of the experiment, suggesting that levels could have eventually surpassed those under the 250 μg/L condition.

Interestingly, TCCs initially increased in the 250 μg/L condition and remained elevated throughout the experiment relative to the undosed control, suggesting that copper acted largely as a nutrient at this concentration. Doses of 4 and 30 μg/L Cu were initially associated with a significant decrease in TCCs (paired *t*-test, *p* < 0.05), but remained comparable to the control for the majority of the experiment.

*M. avium* concentrations, which were only measured in the 11th month of copper dosing, were also highest in the 250 μg/L condition, but were lowest in the 2000 μg/L condition. This suggests that very high doses of copper are likely needed to effectively control *M. avium* and lower doses serve as a nutrient or selector for the organism. One limitation of this study was that *M. avium* was quantified only during the three sequential samplings in the final (11th) month of copper dosing, alongside the *L. pneumophila* ddPCR and 16S rRNA gene sequencing analyses. The original study was focused on *L. pneumophila*, and *M. avium* was additionally quantified at the end point to explore whether control measures mitigating *L. pneumophila* potentially favored *M. avium* growth. Overall, the findings for TCC and *M. avium* were consistent with expectations that copper has a dual role of nutrient and antimicrobial.^2^ Further, a level of copper that is antimicrobial in the short term can behave as a nutrient over a period of months to years as microbial communities adapt to the copper.^16^

Support for this hypothesis was provided in the microbial community analysis. Even though TCCs were elevated in the 250 and 2000 μg/L conditions, microbial diversity was diminished. As richness and evenness declined, a subset of the remaining species were enriched and became dominant in the community (Figures 4 and 6). With a significant decrease in diversity, a few taxa prevailed, making the community vulnerable to stochasticity. This was apparent in terms of the wide variance in microbial community composition among the 2000 μg/L replicates (Figure 5).

Prior research by Song and colleagues using the same municipal tap water supply also assessed the effect of dosed copper on the microbial community in a full-scale water system, but the experimental design differed from the present study in that the dose was incrementally increased from 0, 50, 100, 300, 600, 1200, to 2000 μg/L, while maintaining each concentration for only periods of 4–16 weeks.^9^ Overall Phyla-level trends in *Proteobacteria*, *Deinococcus-Thermus*, *Chloroflexi*, *Acidobacteria*, and *Actinobacteria* were similar to those observed in this work. However, Song and colleagues also observed enrichment over 6 weeks at 1200 μg/L in *Firmicutes,* and enrichment of *Gemmatimonadetes* up through 2000 μg/L. The differences in *Firmicutes* and *Gemmatimonadetes* behavior when comparing the results of this study could potentially be attributed to differences in duration of copper dosing, as Song and colleagues maintained the 2000 μg/L copper dose for 12 weeks,^9^ whereas the microcosms in this study were acclimated at this dose for 11 months.

At 250 μg/L, stochasticity was observed in the concentrations of culturable *Lp*, measurements of which maintained relatively consistent among all replicates at lower copper doses (0, 4, and 30 μg/L total Cu), while almost always being undetectable at 2000 μg/L (Supporting Information, Figure S4). In a prior study investigating the effect of copper pipe in warm premise plumbing systems, *Lp* numbers were initially lowest in the biofilms on copper pipes but eventually increased to the same concentrations as PEX and stainless-steel pipes after two years.^33^ In such studies, it is important to note that copper pipe aging leads to decreasing release of copper into solution, and thus, it is difficult to know if the observed increase in *Lp* was due to lower levels of copper or microbial adaptation.

A synthesis of our results over the past decade using Blacksburg tap water reveals two general responses of experimental plumbing systems with respect to *Lp* inoculation. As one would expect, there are many circumstances in which the system response to inoculated *Lp* is deterministic, in which replicate microcosms behave as true replicates. But here, we report a situation in which stochastic behavior clearly occurred because in some replicate microcosms, *Lp* consistently died off, and in other reactors, it thrived. With the benefit of hindsight, we now recognize similar stochastic behavior for *Lp* among replicate microcosms during at least two prior experiments in our lab.^34,35^ We were reluctant to accept the conclusion that these systems were stochastic, preferring to believe that there were subtle uncontrolled differences between replicates, but we consider the evidence gathered herein to be conclusive. This study demonstrates that even in the most simplistic simulation of premise plumbing, i.e., a glass microcosm containing pipe sections, there are complexities at play that can determine if effluent *Lp* is extremely high or very low.

One interesting discrepancy occurred between the Legiolert MPN/mL and ddPCR gc/mL concentrations, where the Legiolert values were higher. This was unexpected since Legiolert represents culturable *Lp*, whereas ddPCR represents total (live + dead) *Lp* DNA. Prior studies have noted similar discrepancies, possibly due to Legiolert overestimation or DNA losses during filtration and extraction.^36,37^ Recent work also indicates that polycarbonate filters can yield more DNA than mixed-cellulose ester membranes for low-biomass drinking water samples,^38^ suggesting that our concentration method may have contributed to the discrepancy.

Interestingly, the replicate microcosms described herein were deterministic and replicable for measured dimensions of cell counts, mycobacteria, and community analysis, even as they were stochastic for *Lp*. We speculate that the sensitive predator, prey, and parasitic relationships that drive the *Lp* life cycle make it particularly vulnerable to random events that can dictate the trajectory of its ecology in the premise plumbing environment. In a companion paper, we report a discovery that *Neochlamydia* was consistently abundant in the replicate microcosms where *Lp* failed to establish.^39^ Previous research in pure culture settings has shown that amoeba carrying *Neochlamydia* are resistant to *L. pneumophila* infection,^40^ potentially explaining the observed stochastic behavior. If such stochastic behavior for *Lp* is commonplace, as it is in certain other areas of microbial ecology,^41,42^ this discovery has profound implications for experimental design and the interpretation of past data from laboratory and field sampling.^43^ Overall, the findings of this study reveal both hormesis and stochasticity in microbial ecological succession as key mechanisms underlying the observed nonlinear response of *Lp* to copper as a disinfectant in premise plumbing.

## Conclusion

- Over an 11 month copper-dosing period, *M. avium* and TCCs were highest at a Cu dose of 250 μg/L.
- *Lp* growth was stochastic in replicate microcosms and could not be normalized, despite cross-inoculations and reinoculation efforts.
- At the highest copper dose tested (2000 μg/L), both *M. avium* and *Lp* were suppressed, whereas TCCs initially declined but ultimately recovered, indicating adaptation of some community members to high copper.
- Copper can act both as a nutrient and an antimicrobial, with net effect dependent on concentration and other circumstances.
- These findings highlight the importance of optimizing copper-based treatment strategies in premise plumbing to balance microbial suppression with unintended growth, as well as the range of effects from corrosion of copper alloy plumbing.

## Supplementary Material



## Notes

Information regarding ddPCR assays, statistical analysis, initial *Lp* and TCC of the microcosm groups at the end of the acclimation phase, additional TCC information for the 2000 μg/L Cu condition, comparison between Legiolert and ddPCR *Lp* measurements, and the culturable *Lp* for all the copper conditions throughout the study (PDF)
