# Supplementary material for: Influence of Copper Dose on Mycobacterium avium and Legionella pneumophila Growth in Premise Plumbing
Source: ACS ES T Water. 2025 Oct 10;5(11):7005–15. doi: 10.1021/acsestwater.5c00959 (PMC12624713; doi:10.1021/acsestwater.5c00959)
Supplement: Supplementary file 1 [file ew5c00959_si_001.pdf]

Supporting Information

for

Influence of Copper Dose on *Mycobacterium avium* and *Legionella pneumophila* Growth in Premise Plumbing

Authors:

Rania E. Smeltz[‡1,2], Fernando A. Roman Jr*[‡1], Thomas Byrne[‡3], Rachel Finkelstein[‡1,4], Yang Song[1,5], Amy Pruden[1], Marc A. Edwards[1]

[1]Department of Civil and Environmental Engineering, Virginia Tech, Blacksburg, Virginia 24061, United States

[2]Department of Microbiology, University of Alabama at Birmingham, Birmingham, Alabama 35294, United States

[3]Department of Genetics, Bioinformatics, and Computational Biology, Virginia Tech, Blacksburg, Virginia 24061, United States

[4]AECOM, 3101 Wilson Boulevard, Arlington, Virginia 22201, United States

[5]Utilities Department, Town of Cary, 316 N. Academy St., Cary, North Carolina 27513, United States

[‡]These authors are designated as co-first authors and contributed equally to this work

*Corresponding Author froman@vt.edu

Contents:

Supporting Information Table 1: ddPCR assays

Supporting Information Table 2: Statistical Analysis of Total Cell Counts

Supporting Information Table 3: Statistical Analysis of *M. Avium* data

Supporting Information Figure 1: *Legionella pneumophila* and Total Cell Counts in the microcosms at the end of the acclimation phase

Supporting Information Figure 2: Total Cell Counts in 2000 µg/L Cu condition over copper dosing period

Supporting Information Figure 3: Comparison between Legiolert™ and ddPCR targeting *L. pneumophila*

Supporting Information Figure 4: Legiolert™ data collected across study

Supporting Information Table 1. Summary of ddPCR assays.

Target	Supermix	Primers/Probes	Annealing Temperature (°C)	Reference
L. pneumophila	Probe	Forward: AAAGGCATGCAAGACGCTATG	58.9	(Nazarian et al., 2008)[1]
		Reverse: GAAACTTGTTAAGAACGTCTTTCATTTG		
		Probe: FAM-TGGCGCTCAATTGGCTTTAACCGA		
M. avium	Evagreen	Forward: AGAGTTTGATCCTGGCTCAG	59.4	(Wilton et al., 1992)[2]
		Reverse: ACCAGAAGACATGCGTCTTG		

Thermal cycling for *L. pneumophila* amplification consisted of an initial enzyme activation at 95 °C for 10 minutes, followed by 40 cycles of denaturation at 94 °C for 30 seconds and annealing at 58.9 °C for 1 minute. This was followed by a final enzyme deactivation step at 98 °C for 10 minutes, with reactions held at 4 °C thereafter.

Thermal cycling for *M. avium* amplification consisted of an initial enzyme activation at 95 °C for 10 minutes, followed by 40 cycles of denaturation at 95°C for 30 seconds and annealing at 58.9 °C for 1 minute. This was followed by signal stabilization at 4°C for 5 minutes, then at 90 °C for an additional 5 minutes. Reactions were then held at 4°C thereafter.

To evaluate potential PCR inhibition, each sample was initially tested at three concentrations: undiluted, 1:10, and 1:100. Due to low *L. pneumophila* concentrations within the microcosms, undiluted samples were used when targeting *L. pneumophila*. In contrast, *M. avium* was typically quantified using 1:10 dilutions.

Positives were defined as wells with ≥3 positive droplets and more than 10,000 accepted droplets per 20 µL reaction. Based on these criteria, the extraction of 100 µL of DNA from each collected filter, and the corresponding volume of water filtered (~82 mL) from each microcosm, ~1.5 gc/mL was the lowest concentration theoretically detectable under our conditions. In practice, however, the lowest concentrations consistently detected in our dataset were ~2 gc/mL for the *L. pneumophila mip* assay (probe-based) and ~20 gc/mL for the *M. avium* (Evagreen) assay.

Supporting Information Table 2. Tukey contrasts on a linear mixed-effect model of total cell count by copper dosage (0 – 2000 µg/L) on the final month of the copper dosing phase, over three sequential water changes, controlling for repeated microcosm measurements.

Copper	0	4	30	250	2000
0	1	0.980	1	<0.001	<0.001
4	0.980	1	0.978	<0.001	<0.001
30	1	0.978	1	<0.001	<0.001
250	<0.001	<0.001	<0.001	1	0.411
2000	<0.001	<0.001	<0.001	0.411	1

Supporting Information Table 3. Tukey contrasts on a linear mixed-effect model of *M. avium* ddPCR gene copies/mL by copper (0 – 2000 µg/L) on the final month of the copper dosing phase, over three sequential water changes, controlling for repeated microcosm measurements.

Copper	0	4	30	250	2000
0	1	0.998	1	<0.001	0.426
4	0.998	1	1	<0.001	0.251
30	1	1	1	<0.001	0.349
250	<0.001	<0.001	<0.001	1	0.026
2000	0.426	0.251	0.349	0.026	1

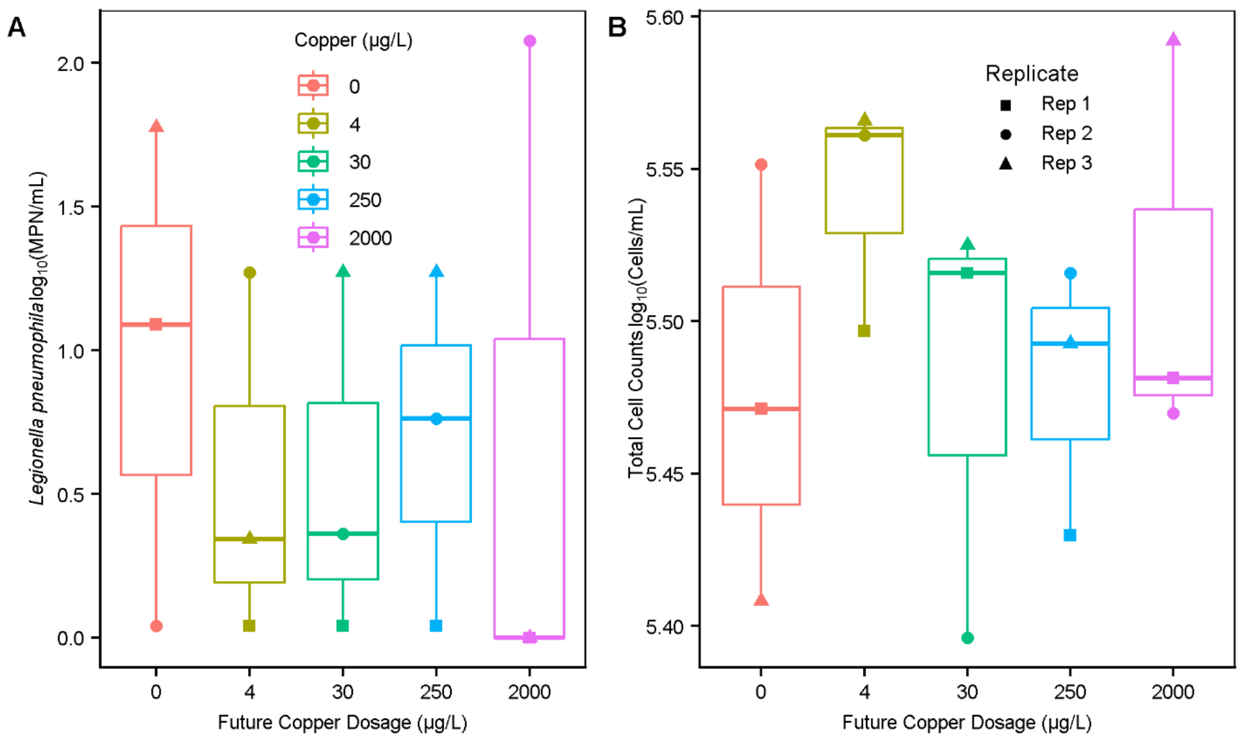

Supporting Information Figure 1. (A) Log *L. pneumophila* and (B) log total cell counts at the end of the acclimation phase, just prior to commencing copper dosing, grouped by the copper dosing they were destined to receive and with the shape corresponding to their replicate number. The data consists of 5 groups (that would later correspond to copper levels received) × 3 microcosms × 1 sampling event = 15 *L. pneumophila* and 15 total cell count data points).

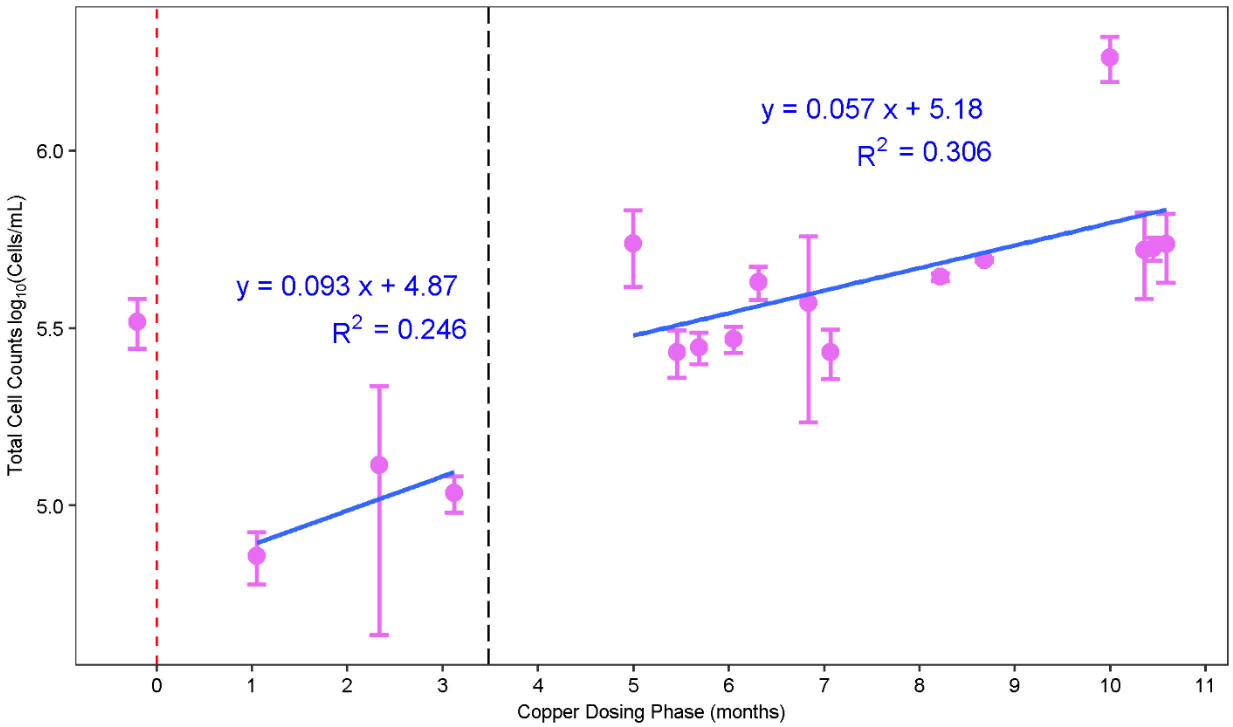

Supporting Information Figure 2. Mean ± standard deviation total cell counts of the 2000 µg/L microcosms throughout the copper-dosing phase (operated in biological triplicate n=3). The dashed red vertical line at Time 0 represents the beginning of copper dosing. The dashed black vertical line at Time ~3.5 represents the point at which all reactors were reinoculated with *L. pneumophila*.

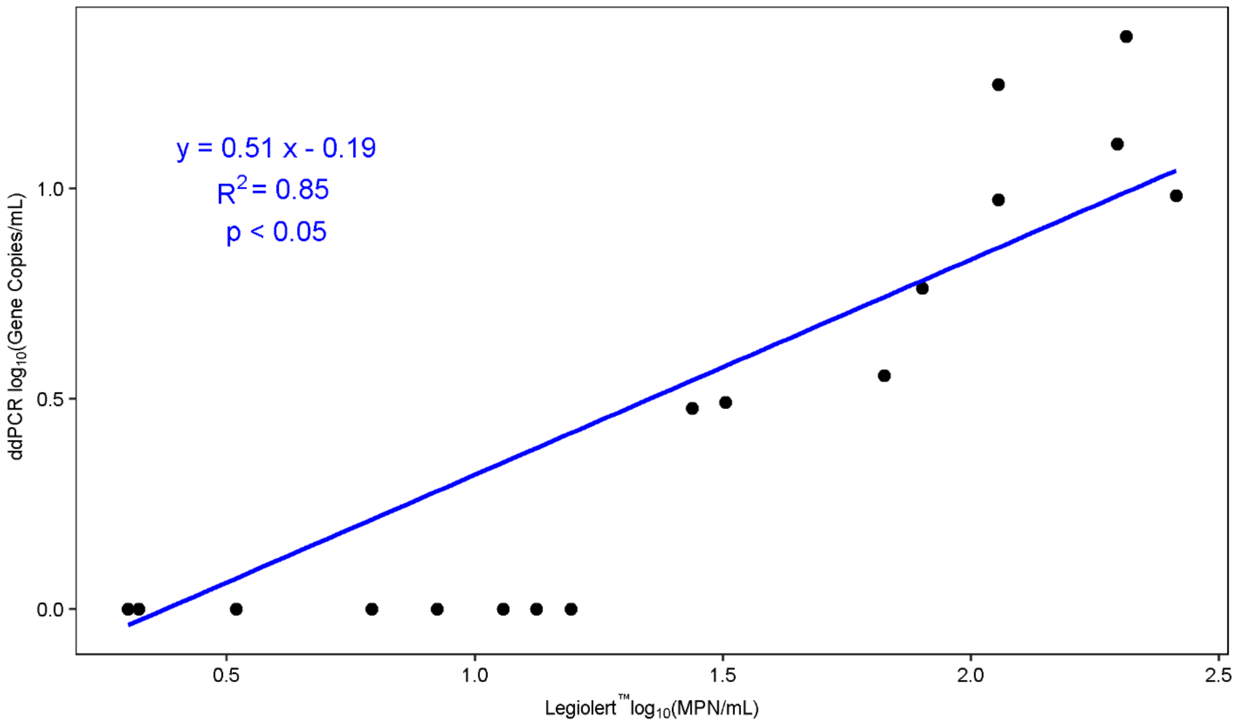

Supporting Information Figure 3. Comparison of Legiolert™ measurements as log(MPN/mL) to ddPCR measurements log(gene copies/mL) of *L. pneumophila* among samples for which both measurements were conducted. The blue line and equation represent the linear regression between the two variables. Data points plotted at a concentration of zero (gc/mL or MPN/mL) represent non-detects. The data consists of 5 copper levels × 3 microcosms (per copper level) × 3 sampling events = 45 data points.

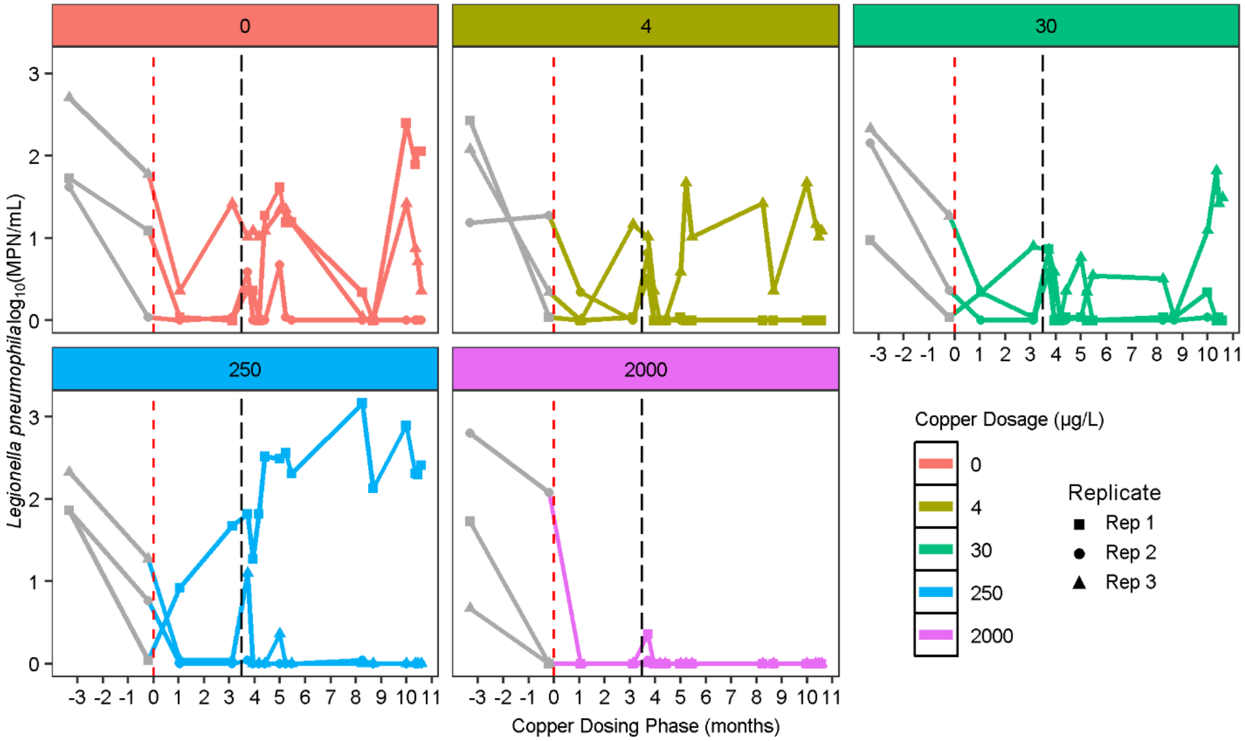

Supporting Information Figure 4. *L. pneumophila* quantification in MPN/mL determined by Legiolert™ tests for the three replicate microcosms dosed with copper over 11 months. The dashed red vertical line at Time 0 represents the time copper dosing began. The dashed black vertical line at ~ 3.5 months represents the point at which all microcosms were reinoculated with water from the original lab system containing *L. pneumophila* from which the microcosms had originally been seeded. The detection limit was 1 MPN/mL. For *L. pneumophila*, points plotted at an MPN/mL of zero indicate non-detects.

References

1. Nazarian, E. J.; Bopp, D. J.; Saylors, A.; Limberger, R. J.; Musser, K. A. Design and Implementation of a Protocol for the Detection of Legionella in Clinical and Environmental Samples. Diagnostic Microbiology and Infectious Disease **2008**, 62 (2), 125–132. DOI:10.1016/j.diagmicrobio.2008.05.004.

2. Wilton, S.; Cousins, D. Detection and Identification of Multiple Mycobacterial Pathogens by DNA Amplification in a Single Tube. Genome Research **1992**, 1 (4), 269–273. DOI:10.1101/gr.1.4.269.
